# Editorial: COVID-19 related olfactory dysfunction: neuropsychiatric, psychological, and cognitive effects

**DOI:** 10.3389/fnins.2023.1306094

**Published:** 2023-11-01

**Authors:** Yang Zhou, Xiaoman Lv, Zhusheng Tang, Dongdong Qin

**Affiliations:** ^1^Key Laboratory of Traditional Chinese Medicine for Prevention and Treatment of Neuropsychiatric Diseases, Yunnan University of Chinese Medicine, Kunming, Yunnan, China; ^2^The First School of Clinical Medicine, Yunnan University of Chinese Medicine, Kunming, Yunnan, China; ^3^Open and Shared Public Science and Technology Service Platform of Traditional Chinese Medicine Science and Technology Resources in Yunnan, Yunnan University of Chinese Medicine, Kunming, Yunnan, China; ^4^College of Pharmacy, Dali University, Dali, Yunnan, China; ^5^The Second School of Clinical Medicine, Yunnan University of Chinese Medicine, Kunming, Yunnan, China; ^6^Yunnan Key Laboratory of Integrated Traditional Chinese and Western Medicine for Chronic Disease in Prevention and Treatment, Yunnan University of Chinese Medicine, Kunming, Yunnan, China

**Keywords:** COVID-19, olfactory dysfunction, affective disorder, cognitive impairment, neuroplasticity

## Introduction

Olfactory dysfunction (OD), also known as loss of smell and hyposmia, is one of the common symptoms of COVID-19 (corona virus disease 2019), with a reported incidence of up to 80–90% (Cardoso et al., 2022). In addition, there are more than 755 million cumulative cases of COVID-19 worldwide, and millions of patients are currently experiencing olfactory and gustatory deficits (Cecchetto et al., 2021; Ohla et al., 2022). Some patients recover in the short term, but a significant portion of patients still suffer from long-term OD even after 2 years of infection with COVID-19 (McWilliams et al., 2022). Furthermore, OD is associated with disturbances in daily life and interpersonal interactions, which are unfavorable to physical and mental health development (Erskine and Philpott, 2020; Elkholi et al., 2021). Individuals with OD often exhibit depressive and anxious states, self-esteem deficits, diminished intensities of affective experiences, and a reduced quality of life (Glezer et al., 2021; Schäfer et al., 2021). This presents novel challenges, emphasizes, and exacerbates unmet needs for adequate access to health care for those COVID-19 patients suffering from OD (Ball et al., 2021).

The short- and long-term neuropsychiatric consequences, as well as the molecular mechanisms, of COVID-19-related OD have not been well studied. Investigating the neuropsychiatric sequelae of OD associated with COVID-19 infection is particularly important to characterize the pathological effects of COVID-19 on brain function and to develop strategies to improve the quality of life and mental health of patients, which is the focus of this Research Topic.

**Figure 1:**
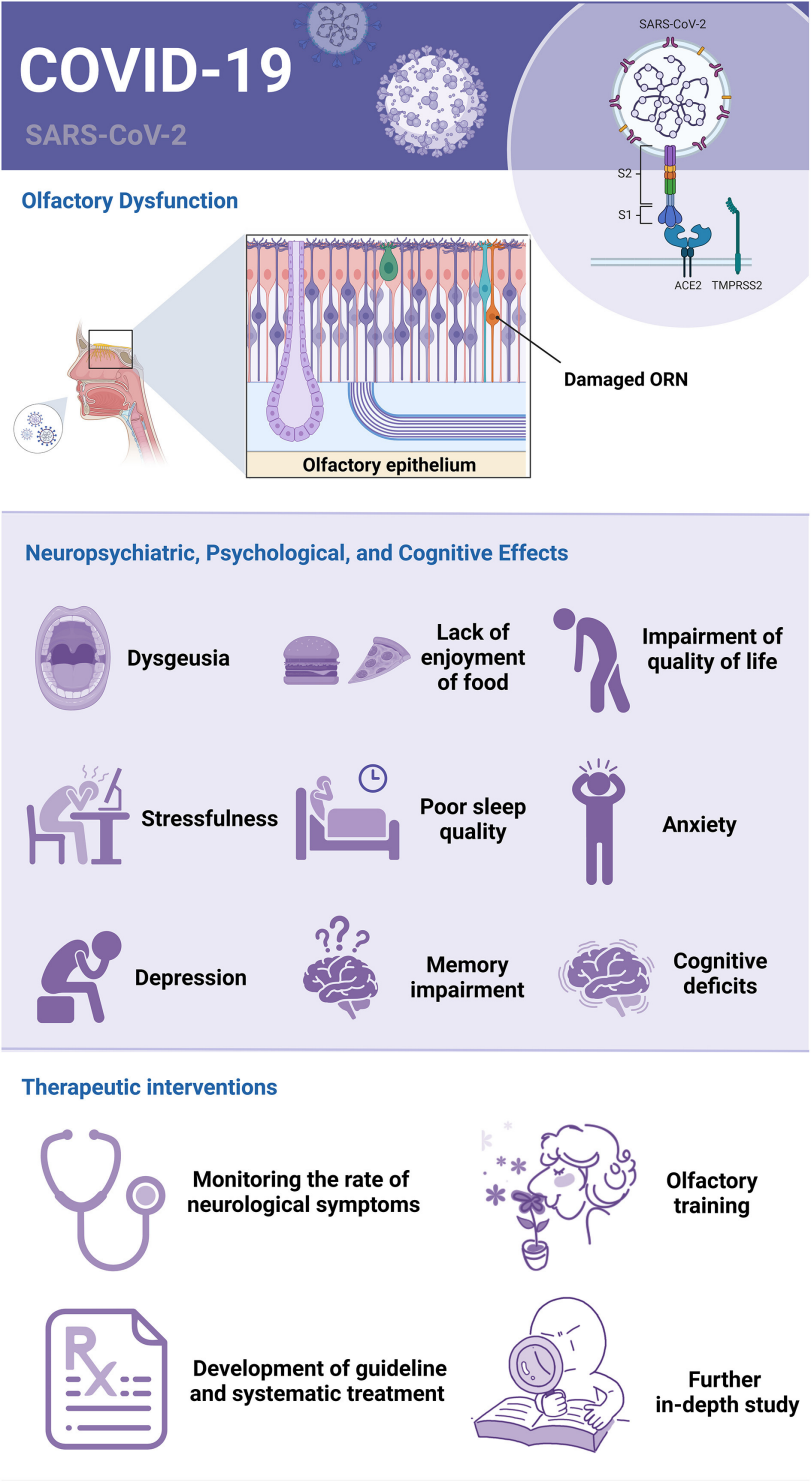
Neuropsychiatric, psychological, and cognitive effects of COVID-19-related olfactory dysfunction and therapeutic interventions. ORN, olfactory receptor neurons.

Within this context, we launched our Research Topic on September 13, 2022, and invited researchers to address the COVID-19 Related Olfactory Dysfunction: Neuropsychiatric, Psychological, and Cognitive Effects both on a personal and a social level. This Research Topic has had the pleasure of receiving diverse and insightful manuscript proposals. Frontiers in Neuroscience published five original articles, four reviews, and one opinion, involving 61 authors from 6 countries, which were contained in Perception Science specialized sections. Based on the objective of each contributed article, we can group them into two major categories, including (1) associations of COVID-19-related OD with neuropsychiatric, psychological, and cognitive consequences; (2) impacts and therapeutic interventions of COVID-19-related OD (Figure 1).

## Associations of COVID-19-related OD with neuropsychiatric, psychological, and cognitive consequences

Llana, Mendez, Zorzo, et al. recruited 42 long-COVID patients and 30 controls. Their objective performance in the dissociative memory system, including associated symptoms of OD, was assessed and compared. The results suggest that COVID-19-induced olfactory deficits might be associated with long-term limbic system dysfunction.

In another 6-month cross-sectional study, Llana, Mendez, Garces-Arilla, et al. enrolled 128 long-COVID participants in Spain, which is the first time to identify correlations between OD and subjective and objective memory scores, general cognitive functioning, and mood disorders. These findings will give easy access to a deeper understanding of the neuropsychological and emotional aspects of the long COVID.

Tai et al. provide a mini-review that summarizes the sense of smell and its neural correlates and discusses possible mechanistic pathways for how COVID-19 affects olfactory function and its damage to the central nervous system, leading to neuropsychiatric symptoms.

In an opinion paper, Kocsis and Pittman-Polletta proposed that damaged non-olfactory inputs generated by the olfactory epithelium are intimately related to cognitive function, and respiratory-related oscillations can be a hidden mechanism for the neuropsychiatric sequelae of OD associated with COVID-19 infection.

Additionally, a captivating scoping review summarized the existing literature to assess the association between neurocognition and olfaction among non-agenarians after COVID-19 infection. Vilarello et al. found that patients with OD had significantly worse cognitive performance, providing a bridge to understanding the neural mechanism underlying the relationship between olfaction and cognition after pericoronitis.

## Impacts and therapeutic interventions of COVID-19-related OD

Yang et al. used VOSviewer to quantitatively analyze and visualize the current research status and development trend of COVID-19-induced OD retrieved from the Web of Science, and finally identified six research hotspots and revealed that the temporal evolution of COVID-19-related OD can be classified into three phases. These results provide effective assessment and intervention strategies for future related studies.

Winter et al. assessed 58 individuals diagnosed with COVID-19 and self-perceived OD using questionnaires and the Sniffin' Sticks extended test battery. The results revealed a positive correlation between qualitative OD and the degree of impairments in daily living. The primary cause of diminished quality of life was the absence of food enjoyment. These outcomes highlight the importance of risk assessment in future clinical studies and the development of new intervention strategies.

A Brazilian cross-sectional survey involved a total of 288 participants with long COVID and self-reported neurological symptoms (anxiety, cognitive impairment, and olfactory disturbances), which were assessed using standardized self-rating scales by Paranhos et al. Anxiety and olfactory disturbances were identified in a high percentage of patients with poor sleep quality. This study recommended that neurological symptoms associated with COVID-19 should be emphasized and monitored in the long term.

Kumari et al. recruited 15 participants with persistent COVID-19-related olfactory and/or gustatory deficits in the United Kingdom. Through the application of a new Camera-Based Visual Feedback Learning Aid (CVFLA) device, the intervention was found to restore olfactory and gustatory deficits associated with COVID-19. The proof-of-concept study also explored possible mechanisms by which CVFLA improved the sense of smell or taste.

In a comprehensive review, Jegatheeswaran et al. summarized the obvious relationships between long-COVID and psychological, neuropsychiatric, and cognitive symptoms, and provided a guideline and theoretical basis for the evaluation of olfactory disorder, and psychophysical effects in patients with COVID-19.

Overall, the original research and review papers in this Research Topic assemble a range of topics that systematically explore the correlations and possible mechanisms of neuropsychiatric, psychological, and cognitive consequences of COVID-19-related OD, which also present the recent and cutting-edge research on the assessment or prevention of long-COVID-related OD, in addition to new therapeutic strategies. This topic paved the way for future research on virus-induced OD-related diseases. We hope that this Research Topic will go some way toward helping researchers around the world to search for more associations between olfactory disorders and neurological impairments, thus helping to refine our understanding of COVID-19 induced multiple comorbidities. Additionally, this topic will aid in guiding public policy formulation.

## Author contributions

YZ: Writing—original draft. XL: Writing—original draft. ZT: Supervision, Writing—review & editing. DQ: Conceptualization, Supervision, Writing—original draft, Writing—review & editing.
